# TACE combined with regorafenib with or without anti-PD-1 therapy for advanced HCC after targeted therapy failure: a multicenter real-world study

**DOI:** 10.3389/fonc.2025.1652319

**Published:** 2025-10-16

**Authors:** Yan Li, Hang Yuan, Quan-Jun Yao, Xiang Geng, Fei Xu, Weijun Fan, Gang Wu, Guang-Shao Cao, Ho-Young Song, Hong-Tao Hu

**Affiliations:** ^1^ Department of Interventional Radiology, The Affiliated Cancer Hospital of Zhengzhou University & Henan Cancer Hospital, Zhengzhou, China; ^2^ Department of Minimal-Invasive Intervention, Cancer Hospital Chinese Academy of Medical Sciences, Beijing, China; ^3^ Department of Minimally Invasive Interventional Radiology, Sun Yat-sen University Cancer Center, Guangzhou, China; ^4^ The First Affiliated Hospital of Zhengzhou University, Zhengzhou, China; ^5^ Department of Intervention, Henan Provincial People’s Hospital, Zhengzhou, China

**Keywords:** anti-PD-1 therapy, combination therapy, hepatocellular carcinoma, regorafenib, transarterial chemoembolization

## Abstract

**Background and objectives:**

Patients with hepatocellular carcinoma (HCC) progressing after targeted therapy face limited treatment options and poor prognosis. Although regorafenib is an established second-line therapy, its combination with locoregional and immunotherapeutic approaches remains insufficiently characterized in real-world settings. This multicenter study evaluated the efficacy and safety of combining transarterial chemoembolization (TACE) with regorafenib and anti-PD-1 therapy in advanced HCC (BCLC B/C) after targeted therapy failure, with a focus on optimizing treatment timing and dosing strategies.

**Methods:**

We conducted a retrospective, multicenter, propensity score-matched study involving 188 HCC patients from five tertiary medical centers between June 2022 and June 2024. Among them, 103 patients received triple therapy (TRP group: TACE combined with regorafenib and PD-1 inhibitors), while 85 received dual therapy (TR group: TACE combined with regorafenib). After propensity score matching (PSM), 64 patients were included in each group. Primary endpoints included progression-free survival (PFS) and overall survival (OS), evaluated per mRECIST v1.1 criteria, with secondary endpoints including objective response rate (ORR), disease control rate (DCR), and treatment-related adverse events (TRAEs). Subgroup analyses examined the effects of regorafenib initiation timing (second-line versus third-line or later) and dosage (80 mg vs 120–160 mg) on PFS.

**Results:**

The triple therapy group demonstrated significantly superior efficacy compared to the dual therapy group. After PSM, the TRP group showed significantly improved median PFS (6.5 vs. 4.6 months) and OS (15.8 vs. 12.1 months), along with significantly higher ORR (32.8% vs. 17.2%) and DCR (.71.9% vs. 51.6%) compared to the TR group. Earlier regorafenib initiation (second-line) was associated with substantially prolonged PFS in both treatment arms (TRP group: 7.2 vs 5.1 months; TR group: 5.1 vs 4.2 months), whereas dosage variations did not significantly affect survival outcomes. TRAEs were comparable between groups except for a higher incidence of rash in the triple therapy group (25.0% vs 6.3%).

**Conclusions:**

The triple combination of TACE, regorafenib, and PD-1 inhibitors demonstrated superior clinical efficacy compared with TACE-regorafenib dual therapy in advanced HCC patients after targeted therapy failure, with optimal outcomes observed following earlier regorafenib initiation and an acceptable safety profile.

## Introduction

Hepatocellular carcinoma (HCC) is one of the most prevalent malignancies worldwide with high mortality (1). Due to its insidious onset, most patients are diagnosed at intermediate-to-advanced stages, becoming ineligible for surgical resection or curative ablation, resulting in a <20% 5-year survival rate (2, 3). Transarterial chemoembolization (TACE) has been widely used for intermediate-advanced HCC (4), with its efficacy well-established in numerous studies. Since 2017, the clinical application of various targeted agents and immunotherapies has progressively transformed the therapeutic landscape (5, 6), making combination therapies the emerging standard approach.

As a second-line targeted agent for HCC, regorafenib has demonstrated prognostic improvement in advanced HCC across multiple clinical studies since the RESORCE study (7, 8). In combination therapies (9), regorafenib plus programmed death-1 (PD-1) inhibitors shows superior efficacy over regorafenib monotherapy for advanced HCC. Similarly, studies (10) indicate enhanced effectiveness when combining regorafenib with TACE versus regorafenib alone as second-line treatment after sorafenib failure. Mechanistically, TACE converts immunologically “cold” HCC tumors into “hot” tumors, thereby potentiating immunotherapeutic responses (11, 12). These findings provide the rationale for combining TACE with regorafenib and PD-1 inhibitors.

Although regorafenib is recommended as standard second-line therapy for advanced HCC in international guidelines (13, 14), its clinical implementation in China remains suboptimal due to high economic burden and significant adverse events, particularly regarding timing of initiation and dosing regimens. This multicenter retrospective real-world study evaluates the efficacy and safety of TACE combined with regorafenib, with or without PD-1 inhibitors, in patients with intermediate-advanced HCC following progression on targeted therapies.

## Material and methods

### Patients

This retrospective study analyzed 309 patients with advanced HCC who underwent TACE combined with regorafenib between June 2022 and June 2024 at five tertiary hospitals. All patients demonstrated confirmed radiological progression following prior targeted systemic therapy prior to initiating the TACE-regorafenib combination or non combination of PD-1 therapy.

The inclusion criteria were: (1) age 18–80 years; (2) pathologically or clinically confirmed HCC; (3) ECOG performance status ≤2; (4) BCLC stage B or C disease; (5) Child-Pugh class A or B liver function; (6) disease progression occurred after treatment with targeted agents (e.g., sorafenib, lenvatinib, apatinib, or bevacizumab); (7) presence of ≥1 measurable target lesion(s) on imaging (including multinodular HCC); (8) no previous immunotherapy; and (9) complete clinical follow-up records. Key exclusion criteria included: (a) current or historical malignancies other than HCC; (b) severe systemic comorbidities including significant organ dysfunction or coagulopathy; (c) receipt of local therapies other than TACE and curative ablation (e.g., HAIC, radiation therapy); and (d) follow-up duration <3 months.

### TACE procedure

The TACE procedures were performed via the femoral approach under local anesthesia by two experienced interventional radiologists. After routine angiography with a 5F RH catheter (Terumo, Tokyo, Japan), superselective cannulation of the tumor-feeding arterial branch was achieved using a microcatheter (Hengrui, Jiangsu, China). Chemotherapy agents (raltitrexed, oxaliplatin, and fluorouracil) were administered first, followed by an emulsion of doxorubicin (Haizheng Pharmaceutical, Taizhou, China) and lipiodol (Hengrui, Jiangsu, China). Finally, 560–710 μm gelatin sponge particles (ALICON, Hangzhou, China) were injected until near-stasis of blood flow was achieved. The doses of lipiodol and chemotherapeutic agents were adjusted based on liver function, tumor burden, and body surface area.

### Regorafenib and PD-1 inhibitors

All patients received oral regorafenib (Bayer AG, Germany) at 80–160 mg once daily in 4-week cycles (3 weeks on/1 week off). PD-1 inhibitors (including camrelizumab, sintilimab, tislelizumab, or pembrolizumab) were administered intravenously at 200 mg every 3 weeks. Adverse events (AEs) were assessed according to the National Cancer Institute Common Terminology Criteria for Adverse Events (NCI-CTCAE). For patients experiencing grade ≥3 treatment-related adverse events (TRAEs), appropriate management was implemented, including dose reduction, treatment delay, or discontinuation when necessary.

### Follow-up and evaluation

Patients were followed every 4–6 weeks after combination therapy for ≥6 months, with the final follow-up in February 2025. Follow-up assessments included survival status, imaging examinations (contrast-enhanced CT/MRI), laboratory tests, and AE documentation. Treatment response was evaluated by ≥2 senior radiologists according to the modified Response Evaluation Criteria in Solid Tumors (mRECIST) (15) and RECIST version 1.1. The primary endpoint was progression-free survival (PFS), with secondary endpoints including overall survival (OS), objective response rate (ORR), disease control rate (DCR), and AEs. PFS was defined as the time from treatment initiation to death or first documented progression, while OS represented the time from treatment initiation to death.

### Statistical analysis

Propensity score matching (PSM) was employed to mitigate selection bias and balance baseline characteristics between groups. Continuous variables are presented as mean ± standard deviation, while categorical variables are expressed as frequencies (percentages). OS and PFS were analyzed using Kaplan-Meier curves with log-rank tests. Univariate and multivariate Cox proportional hazards regression models were utilized to identify independent prognostic factors for PFS, with a P-value < 0.05 considered statistically significant. All statistical analyses were performed using R software (version 4.3.2; R Foundation for Statistical Computing, Vienna, Austria. URL: https://www.R-project.org/).

## Result

### Patient characteristics

**Figure 1:**
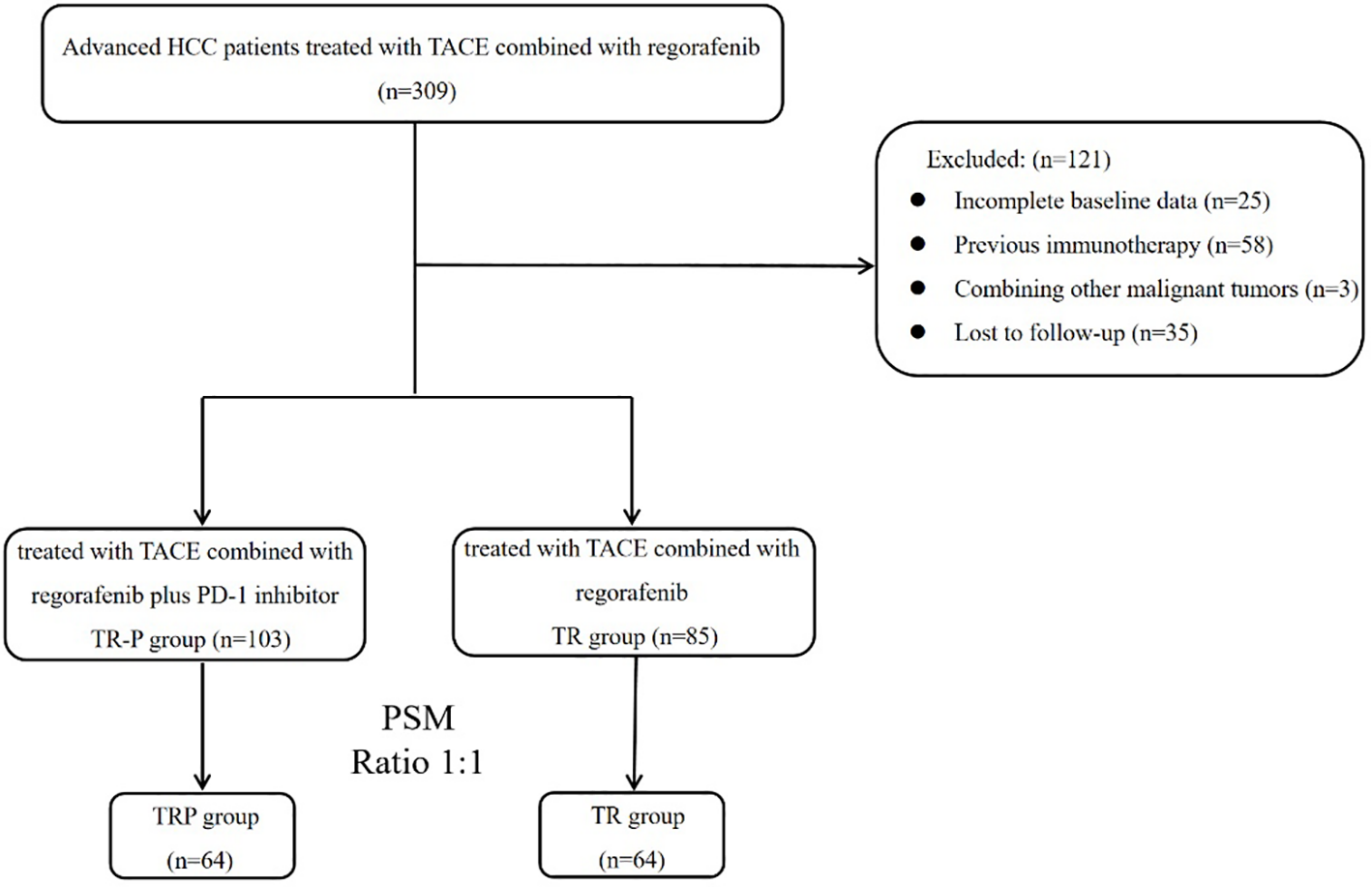
Patient selection flowchart.

After screening based on the inclusion/exclusion criteria, 188 patients were enrolled (**Figure 1**). Based on whether PD-1 inhibitors were administered concurrently, patients were stratified into the TRP group (TACE combined with regorafenib and PD-1 inhibitors; n=103) and the TR group (TACE combined with regorafenib; n=85). Gender distribution, presence of cirrh, osis and time of regorafenib initiation differed significantly between the two groups (P<0.05). After PSM, 64 pairs were generated. There were no significant differences in baseline characteristics between the two groups after matching (**Table 1**).

**Table 1:** Baseline characteristics of the two groups before and after PSM.

Variable	Before PSM			After PSM		
	TR (n=85)	TRP (n=103)	*P* value	TR (n=64)	TRP (n=64)	*P* value
Age (years)	54.8±10.2	53.4±7.9	0.277	53.5±9.9	54.2±7.4	0.681
Gender			**0.039**			0.694
Male	64(75.3)	63(61.2)		47(73.4)	45(70.3)	
Female	21(24.7)	40(38.8)		17(26.6)	19(29.7)	
Cirrhosis			**0.041**			0.795
Yes	75(88.2)	79(76.7)		55(85.9)	56(87.5)	
No	10(11.8)	24(23.3)		9(14.1)	8(12.5)	
Viral Hepatitis			0.336			0.811
Yes	73(85.9)	83(80.6)		54(84.4)	53(82.8)	
No	12(14.1)	20(19.4)		10(15.6)	11(17.2)	
ECOG			0.594			0.843
0-1	64(75.3)	74(71.8)		46(71.9)	47(73.4)	
2	21(24.7)	29(28.2)		18(28.1)	17(26.6)	
BCLC			0.279			0.457
B	25(29.4)	38(36.9)		24(37.5)	20(31.3)	
C	60(70.6)	65(63.1)		40(62.5)	44(68.8)	
Child-Pugh			0.127			0.432
A	67(78.8)	71(68.9)		48(75.0)	44(68.8)	
B	18(21.2)	32(31.1)		16(25.0)	20(31.3)	
AFP			0.298			0.152
<400 ng/ml	34(40.0)	49(47.6)		23(35.9)	31(48.4)	
≥400 ng/ml	51(60.0)	54(52.4)		41(64.1)	33(51.6)	
Timing of regorafenib initiation			**0.039**			0.857
2L	58(68.2)	55(53.4)		39(60.9)	38(59.4)	
3L+	27(31.8)	48(46.6)		25(39.1)	26(40.6)	
Regorafenib dosage			0.533			0.858
80mg	44(51.8)	58(56.3)		37(57.8)	36(56.3)	
120-160mg	41(48.2)	45(43.7)		27(42.2)	28(43.7)	
Number of intrahepatic tumors			0.917			0.811
≤3	12(14.1)	14(13.6)		10(15.6)	11(17.2)	
>3	73(85.9)	89(86.4)		54(84.4)	53(82.8)	
Sum of diameters of intrahepatic tumors			0.107			0.651
≤10cm	14(16.5)	27(26.2)		13(20.3)	11(17.2)	
>10cm	71(83.5)	76(73.8)		51(79.7)	53(82.8)	
Portal vein tumor thrombus			0.877			0.582
Yes	48(56.5)	57(55.3)		39(60.9)	42(65.6)	
No	37(43.5)	46(44.7)		25(39.1)	22(34.4)	
Distant metastases			0.446			0.579
Yes	36(42.4)	38(36.9)		21(32.8)	24(37.5)	
No	49(57.6)	65(63.1)		43(67.2)	40(62.5)	
Prior TACE			0.127			0.710
Yes	61(71.8)	63(61.2)		43(67.2)	41(64.1)	
No	24(28.2)	40(38.8)		21(32.8)	23(35.9)	
Prior surgery or curative ablation			0.952			0.856
Yes	54(63.5)	65(63.1)		40(62.5)	39(60.9)	
No	31(36.5)	38(36.9)		24(37.5)	25(39.1)	
Number of TACE after combination therapy	3.3±1.7	2.9±1.9	0.502	3.2±1.5	3.0±1.7	0.766

TRP, TACE combined with regorafenib and PD-1 inhibitors; TR, TACE combined with regorafenib; PSM, propensity score matching; ECOG, Eastern Cooperative Oncology Group; BCLC, Barcelona Clinic Liver Cancer; 2L, second-line therapy; 3L+, third-line or later therapy. Bold values represent p-values < 0.05, indicating statistically significant differences between the two groups.

### Tumor response and patient survival

**Figure 2:**
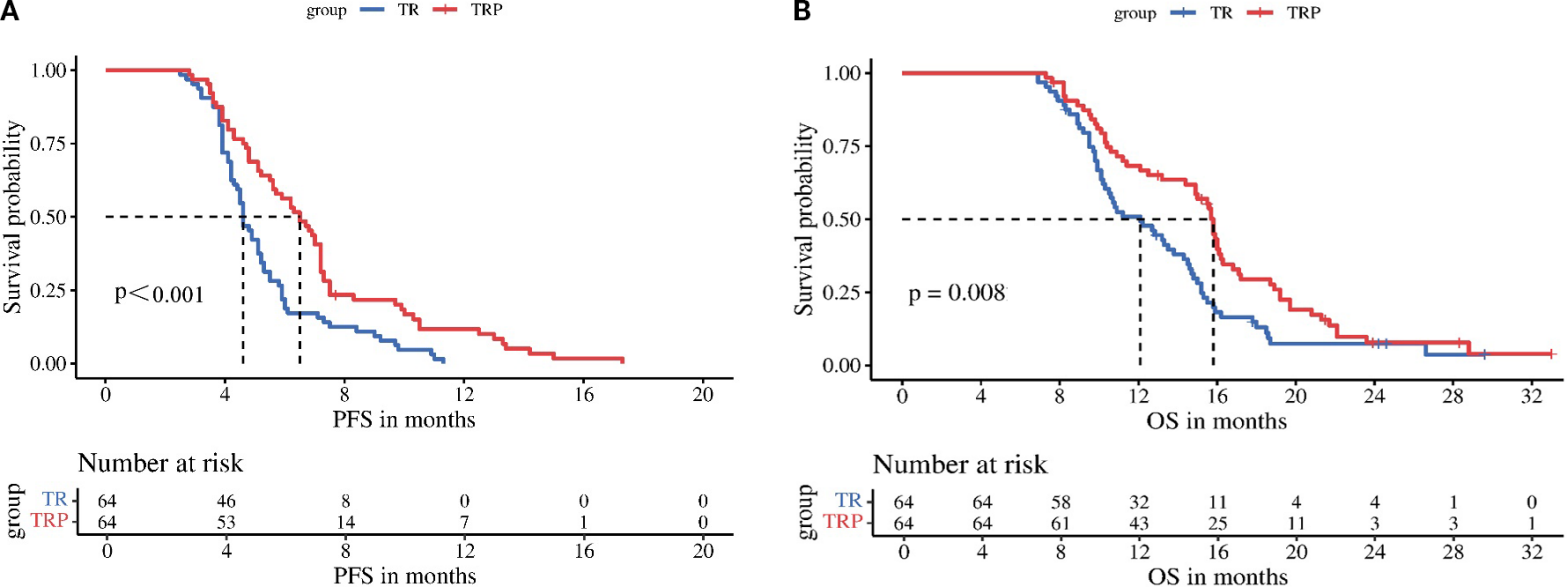
Kaplan-Meier analysis of progression-free survival **(A)** and overall survival **(B)** between the two groups.

Tumor response was evaluated according to both mRECIST and RECIST v1.1 criteria before and after PSM. Before PSM, no significant difference in ORR was observed between the TRP and TR groups by either mRECIST (28.2% vs 16.5%, P = 0.058) or RECIST v1.1 (15.5% vs 9.4%, P = 0.211). However, the DCR was significantly higher in the TRP group with both evaluation methods (mRECIST: 66.0% vs 49.4%, P = 0.021; RECIST v1.1: 59.2% vs 44.7%, P = 0.047). After PSM, the TRP group demonstrated consistently superior outcomes across both criteria. By mRECIST, the TRP group showed significantly improved ORR (32.8% vs 17.2%, P = 0.041) and DCR (71.9% vs 51.6%, P = 0.018). Similarly, when assessed by RECIST v1.1, the TRP group maintained a higher DCR (60.9% vs 40.6%, P = 0.018) and a numerically increased ORR (23.4% vs 10.9%, P = 0.061), though the latter did not reach statistical significance. These results consistently indicate enhanced tumor response with the TRP regimen compared to TR alone, particularly after adjustment for baseline characteristics (**Tables 2A**, **B**). After PSM, survival outcomes were significantly improved in the TRP group, with median PFS of 6.5 vs. 4.6 months (P < 0.001) and median OS of 15.8 vs. 12.1 months (P = 0.008) (**Figure 2**).

**Table 2A:** Tumor responses based on mRECIST before and after PSM.

Tumor response	Before PSM		*P* value	After PSM		*P* value
	TRP (n=103)	TR (n=85)		TRP (n=64)	TR (n=64)	
CR	3	1		2	1	
PR	26	13		19	10	
SD	39	28		25	22	
PD	35	43		18	31	
ORR	29 (28.2%)	14 (16.5%)	0.058	21 (32.8%)	11 (17.2%)	**0.041**
DCR	68 (66.0%)	42 (49.4%)	**0.021**	46 (71.9%)	33 (51.6%)	**0.018**

TRP, TACE combined with regorafenib and PD-1 inhibitors; TR, TACE combined with regorafenib. Based on mRECIST, CR, complete response; PR, partial response; SD, stable disease; PD, progressive disease; ORR, objective response rate; DCR, disease control rate. Bold values represent p-values < 0.05, indicating statistically significant differences between the two groups.

**Table 2B:** Tumor responses based on RECIST v1.1 before and after PSM.

Tumor response	Before PSM		*P* value	After PSM		*P* value
	TRP (n=103)	TR (n=85)		TRP (n=64)	TR (n=64)	
CR	0	0		0	0	
PR	16	8		15	7	
SD	45	30		24	19	
PD	42	47		25	38	
ORR	16 (15.5%)	8(9.4%)	0.211	15 (23.4%)	7 (10.9%)	0.061
DCR	61 (59.2%)	38 (44.7%)	**0.047**	39 (60.9%)	26 (40.6%)	**0.018**

TRP, TACE combined with regorafenib and PD-1 inhibitors; TR, TACE combined with regorafenib. Based on RECIST v1.1. CR, complete response; PR, partial response; SD, stable disease; PD, progressive disease; ORR, objective response rate; DCR, disease control rate. Bold values represent p-values < 0.05, indicating statistically significant differences between the two groups.

**Figure 3:**
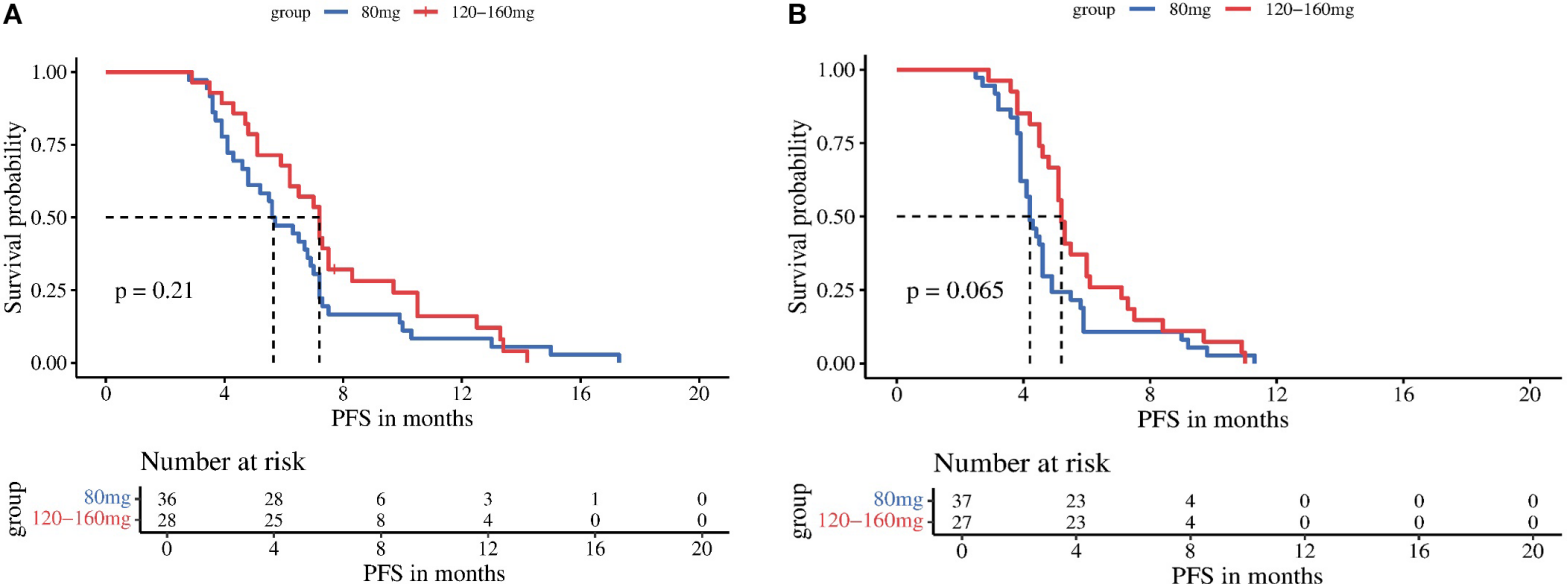
Kaplan-Meier analysis of progression-free survival in TRP group **(A)** and TR group **(B)**, stratified by regorafenib dosage (80 mg vs 120-160 mg).

**Figure 4:**
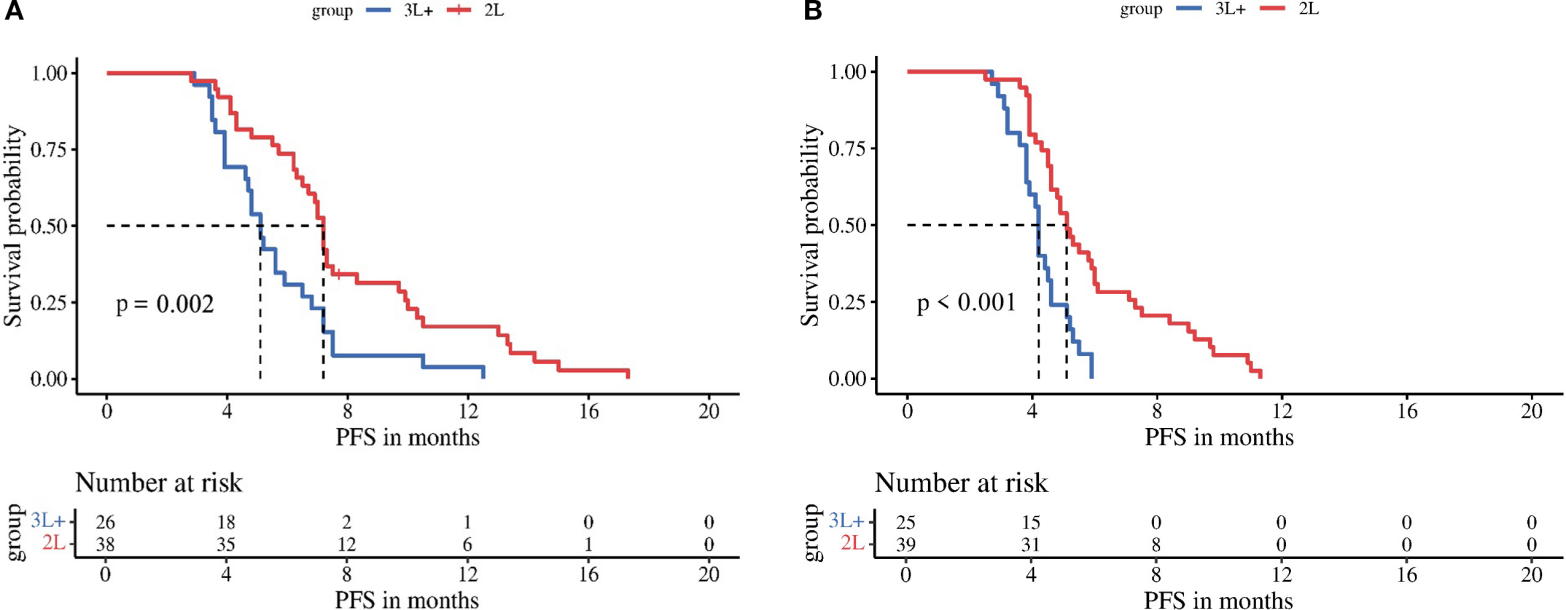
Kaplan-Meier analysis of progression-free survival in TRP group **(A)** and TR group **(B)**, stratified by timing of regorafenib initiation (second-line therapy vs third-line or later therapy).

Subgroup analyses stratified by regorafenib dosage (80 mg vs 120–160 mg) revealed no significant differences in mPFS between dose groups for either treatment arm: the TRP group (5.6 vs 7.2 months, P = 0.210) and the TR group (4.2 vs 5.2 months, P = 0.065) (**Figure 3**). In contrast, stratification by timing of regorafenib initiation (second-line therapy vs third-line or later therapy) demonstrated significantly longer mPFS with earlier treatment initiation in both arms: the TRP group (7.2 vs 5.1 months, P = 0.002) and the TR group (5.1 vs 4.2 months, P < 0.001) (**Figure 4**).

### Prognostic factors associated with PFS

**Figure 5:**
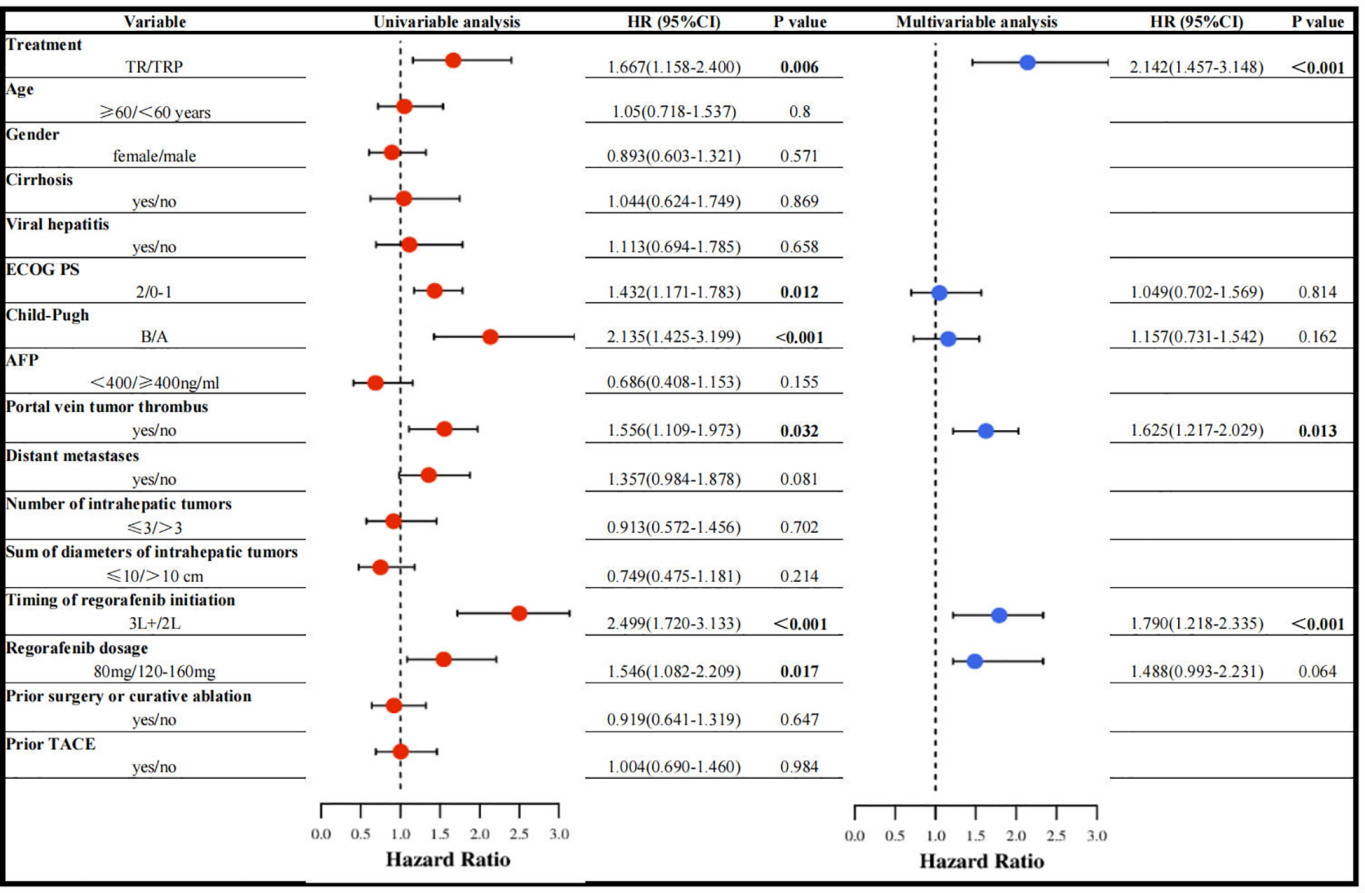
Univariable and multivariable analysis of prognostic indicators for PFS.

Univariable Cox regression analysis revealed that treatment regimen, ECOG PS, Child-Pugh class, portal vein tumor thrombus (PVTT), timing of regorafenib initiation, and regorafenib dosage were associated with PFS. In multivariable analysis, three factors emerged as independent predictors: treatment regimen, PVTT, and timing of regorafenib initiation (**Figure 5**).

### Treatment safety

Overall, 121 patients (94.5%) experienced TRAEs of varying grades (**Table 3**), with no deaths attributed to TRAEs. The most common TRAEs in both groups included fatigue, decreased appetite, fever, nausea, abdominal pain, diarrhea, abnormal liver function, and hypertension. All symptoms improved with supportive care. Aside from rash, there were no significant differences in TRAEs between the two groups. Rash occurred more frequently in the TRP group (25.0% vs 6.3%, P< 0.05).

**Table 3:** Treatment-related adverse events in the two groups.

Adverse events	Any grade			Grade 3-4		
	TRP (n=64)	TR (n=64)	*P* value	TRP (n=64)	TR (n=64)	*P* value
Fatigue	35(54.7%)	30(46.9%)	0.377	8(12.5%)	10(15.6%)	0.611
Decreased appetite	25(39.1%)	29(45.3%)	0.474	15(23.4%)	13(20.3%)	0.669
Fever	19(29.7%)	23(35.9%)	0.451	2(3.1%)	1(1.6%)	1
Nausea	17(26.6%)	22(34.4%)	0.337	4(6.3%)	2(3.1%)	0.676
Abdominal pain	17(26.6%)	15(23.4%)	0.683	5(7.8%)	6(9.4%)	0.752
Diarrhea	14(21.9%)	11(17.2%)	0.504	4(6.3%)	3(4.7%)	1
Elevated AST/ALT	29(45.3%)	26(40.6%)	0.592	6(9.4%)	5(7.8%)	0.752
Hand–foot skin reaction	23(35.9%)	27(42.2%)	0.469	6(9.4%)	8(12.5%)	0.571
Hypertension	21(32.8%)	18(28.1%)	0.565	13(20.3%)	15(23.4%)	0.669
Proteinuria	11(17.2%)	13(20.3%)	0.651	2(3.1%)	1(1.6%)	1
Rash	16(25.0%)	4(6.3%)	**0.003**	3(4.7%)	0	0.244
Hypothyroidism	7(10.9%)	3(4.7%)	0.323	0	0	

TRP, TACE combined with regorafenib and PD-1 inhibitors; TR, TACE combined with regorafenib. Based on CTCAE v5.0, ALT, alanine aminotransferase; AST, aspartate aminotransferase. Bold values represent p-values < 0.05, indicating statistically significant differences between the two groups.

## Discussion

The multicenter, retrospective, real-world study demonstrated that both the triple-therapy regimen (TACE combined with regorafenib and PD-1 inhibitors; mPFS: 6.5 months, mOS: 15.8 months) and the dual-therapy regimen (TACE plus regorafenib; mPFS: 4.6 months, mOS: 12.1 months) exhibited favorable efficacy and safety in patients with advanced HCC after progression on targeted therapy. The triple-therapy regimen showed superior clinical outcomes, with a significant improvement in mPFS.

The landmark RESORCE trial (7) established regorafenib’s efficacy as second-line therapy, showing significant improvements over placebo in advanced HCC (mPFS: 3.1 vs 1.5 months; mOS: 10.6 vs 7.8 months; 38% mortality risk reduction). Our results extend these findings, with both the TRP (mPFS: 6.5 months; mOS: 15.8 months) and TR (mPFS: 4.6 months; mOS: 12.1 months) regimens outperforming RESORCE’s benchmarks, confirming the superiority of combination approaches over regorafenib monotherapy. These observations align with contemporary studies: Zou et al. reported 6.3-month mPFS and 19.7-month mOS with TACE plus regorafenib and PD-1 inhibitors post-sorafenib (16), while Zheng et al. documented 7-month mPFS and 11-month mOS using TACE plus regorafenib and camrelizumab (17). A multicenter retrospective analysis (18) further validated the survival advantage of regorafenib and PD-1 inhibitor combinations (mPFS: 9 months; mOS: 14 months). The observed clinical benefits likely derive from multimodal synergy. TACE initiates tumor ischemia and hypoxia, leading to elevated HIF-1α and subsequent VEGF upregulation through promoter binding-mediated transcriptional activation (19–21). This pro-angiogenic response is effectively countered by regorafenib, a multi-kinase inhibitor that selectively blocks VEGF signaling, thereby suppressing tumor neovascularization and enhancing TACE’s therapeutic efficacy. Concurrently, TACE-induced immunogenic cell death releases tumor antigens while reducing immunosuppressive factors, creating a permissive environment for PD-1 inhibitor activity (22). Regorafenib further augments this synergy by normalizing the tumor vasculature and immune microenvironment (23), while its inhibition of CSF1R and modulation of VEGFR2/3 promotes macrophage reprogramming and CD8+ T-cell activation, establishing a robust antitumor immune response.

The study validates PVTT as an independent predictor of PFS, corroborating previous research (24–26). PVTT exemplifies hepatocellular carcinoma’s most aggressive phenotype, characterized by tumor cells overexpressing epithelial-mesenchymal transition (EMT) markers to attain metastatic competence (27). These cells disseminate via portal flow—either anterograde or retrograde—establishing intrahepatic and extrahepatic metastases that fuel disease progression. The thrombus further exacerbates tumor advancement through dual mechanisms: mechanical obstruction of portal circulation and microenvironmental reprogramming that stimulates tumor necrosis factor-alpha (TNF-α) secretion, collectively impairing hepatic function while accelerating oncologic aggression through inflammatory cascades.

Notably, the study identifies the timing of regorafenib initiation as a critical prognostic factor. Multivariable analysis confirmed it as an independent predictor for PFS, with significantly longer mPFS observed in both the TRP group (7.2 vs 5.1 months) and TR group (5.1 vs 4.2 months) when administered as second-line versus third-line or later therapy, underscoring the clinical value of timely regorafenib use post-targeted therapy failure. These findings align with the REFINE study (28, 29),—a large-scale real-world analysis of 1,005 unresectable HCC patients—which demonstrated superior survival with second-line regorafenib (17.4 months) compared to third-line or later use (9.7 months), further validating the survival benefit of early intervention. Studies by Zhu Kangshun et al. (30) and Ye Mao et al. (31) investigated the efficacy of regorafenib combined with PD-1 inhibitors as second-line therapy in advanced HCC. Their studies reported an mPFS of 5.6–5.9 months, mOS of 12.9–13.4 months, ORR of 36.2%, and DCR of 66.7%. Although our TRP group included additional TACE treatment, the outcomes (ORR 32.8%, DCR 71.9%, mPFS 6.5 months, mOS 15.8 months) were comparable to those of the dual-therapy regimens mentioned above. We speculate that this similarity may be attributed to the fact that 40.6% of patients in our TRP group received regorafenib as third-line or later therapy, which may have attenuated its efficacy. This observation further underscores the importance of initiating regorafenib in a timely manner at the second-line setting.

The study demonstrated that regorafenib dosage (80 mg vs 120–160 mg) did not significantly affect PFS in either the TRP or TR group, with multivariable analysis confirming dosage was not an independent prognostic factor for PFS. These results suggest that for patients with relatively poor baseline status, clinicians may consider initiating regorafenib at 80 mg, with subsequent flexible dose adjustments guided by individual tolerance—an approach justified by the minimal observed impact of dosage on survival outcomes. This flexible dosing approach maintains therapeutic efficacy while potentially reducing adverse events, thereby improving treatment adherence and enabling long-term therapy continuation.

The study demonstrated that both treatment regimens exhibited favorable safety profiles. While the addition of PD-1 inhibitors increased the incidence of certain immune-related adverse events (irAEs), it did not significantly elevate the risk of grade 3–4 TRAEs. The most common adverse reactions in both groups were fatigue (54.7% vs 46.9%), decreased appetite (39.1% vs 45.3%), and transaminase elevation (45.3% vs 40.6%), consistent with previous reports (10, 32). Notably, the TRP group showed significantly higher rates of rash (25.0% vs 6.3%, P = 0.003) and a trend toward increased thyroid dysfunction (10.9% vs 4.7%), highlighting the need for vigilant monitoring of cutaneous and endocrine toxicities during combination immunotherapy. Importantly, with standardized monitoring and management protocols, the triple therapy regimen (TACE plus regorafenib and PD-1 inhibitor) maintained acceptable safety parameters, representing a viable new therapeutic option for clinical practice.

The study has several limitations. First, as a multicenter retrospective analysis, although PSM was employed to balance baseline characteristics between groups, the potential patient selection bias cannot be completely avoided. Second, a variety of different PD-1 inhibitors were included in this study, and the consistency of efficacy may not be guaranteed.

## Conclusions

For patients with advanced HCC progressing after targeted therapy, the combination of TACE, regorafenib and PD-1 inhibitors demonstrated superior efficacy compared to TACE plus regorafenib alone, with manageable toxicity. Earlier initiation of regorafenib was associated with greater clinical benefit.

## Data Availability

The raw data supporting the conclusions of this article will be made available by the authors, without undue reservation.
